# Addressing Current Challenges in Poultry Meat Safety: Development of a Cultivation and Colony Hybridization Approach to Recover Enterotoxigenic *Clostridium perfringens* from Broiler Chicken Carcasses

**DOI:** 10.3390/pathogens13010030

**Published:** 2023-12-28

**Authors:** Rosette Kakese Mukosa, Alexandre Thibodeau, John Morris Fairbrother, William Thériault, Marie-Lou Gaucher

**Affiliations:** 1 Research Chair in Meat Safety, Department of Pathology and Microbiology, Faculty of Veterinary Medicine, University of Montreal, Montreal, QC J2S 2M2, Canada; rosette.kakese.mukosa@umontreal.ca (R.K.M.); alexandre.thibodeau@umontreal.ca (A.T.); william.p.theriault@umontreal.ca (W.T.); 2 Research Group on Infectious Diseases in Animal Production, Department of Pathology and Microbiology, Faculty of Veterinary Medicine, University of Montreal, Montreal, QC J2S 2M2, Canada; john.morris.fairbrother@umontreal.ca; 3 Swine and Poultry Infectious Diseases Research Centre (CRIPA-FRQNT), Faculty of Veterinary Medicine, University of Montreal, Montreal, QC J2S 2M2, Canada; 4 OIE Reference Laboratory for *Escherichia coli*, Faculty of Veterinary Medicine, University of Montreal, Montreal, QC J2S 7C6, Canada

**Keywords:** broiler chicken carcass rinsate, filtration, direct plating, colony hybridization, *cpe* gene, enterotoxigenic *Clostridium perfringens*

## Abstract

Enterotoxigenic *Clostridium perfringens* is one of the main causes of foodborne illness in Canada. The use of a conventional bacterial culture approach to isolate enterotoxigenic *C. perfringens* from poultry meat is common. This approach is based on the phenotype attributable to a double hemolysis phenomenon, whereas few enterotoxigenic strains of *C. perfringens* produce it, which further complicates the study of the reservoirs of this important pathogen. The objectives of the current study were to validate the ability of a digoxigenin-labeled probe to detect the *C. perfringens cpe* gene and to validate the use of either a filtration or a direct plating approach, combined with colony hybridization to detect enterotoxigenic *C. perfringens*. Pure DNA and pure colonies of enterotoxigenic *C. perfringens* and broiler chicken carcass rinsate samples were subjected to colony hybridization. The results showed that the synthesized DNA probe can detect the *cpe* gene from both DNA and pure colonies of enterotoxigenic *C. perfringens*, and from colonies grown from carcass rinsates artificially contaminated with enterotoxigenic *C. perfringens*. Our study suggests that this isolation method is a promising tool for a better understanding of the epidemiology of this zoonotic pathogen.

## 1. Introduction

*Clostridium perfringens*, a Gram-positive, rod-shaped, anaerobic microorganism is found in soils, wastewaters, foods, feces, and is part of the normal intestinal microbiota of warm-blooded animals and humans [1,2]. In addition to being capable of forming spores, the virulence of this microorganism has been historically attributed to its production of about 20 potent toxins, responsible for histotoxic, neurological, and intestinal infections [3,4]. For many years, *C. perfringens* has been classified into five toxinotypes, from A to E, based on the carriage of the four main toxin-encoding genes *plc* (alpha toxin or CPA), *cpb* (beta toxin or CPB), *iap* (iota toxin or ITX), and *etx* (epsilon toxin or ETX) [5]. Recently, this classification system has been revised to incorporate two new complementary types, namely, toxinotypes F and G, respectively representing strains of *C. perfringens* producing the enterotoxin CPE and those carrying a necrotic enteritis B-like toxin (NetB), which is recognized as a major virulence attribute of avian necrotic enteritis-causing *C. perfringens* [4]. This recently revised classification scheme recognizes types B, C, and D strains of *C. perfringens* as the main cause of enteric diseases in animals and restricts type A strains to the gas gangrene-causing isolates, redirecting *C. perfringens* carrying both the *plc* and *cpe* genes to the new toxinotype F [4]. Formerly identified as type A *cpe*-positive *C. perfringens*, strains belonging to type F are now recognized as enterotoxigenic *C. perfringens* producing an enterotoxin (CPE) which is a member of the aerolysin pore-forming toxin family, and has been associated with human gastrointestinal diseases such as food poisoning, antibiotic-associated diarrhea (AAD), sporadic diarrhea (SD), and nosocomial diarrheal illness [6].

Each year, the self-limiting foodborne diarrhea attributed to enterotoxigenic *C. perfringens* afflicts approximately 176,000, 1,000,000, and 80,000 people in Canada, the United States, and the United Kingdom, respectively, resulting in an economic burden of USD 382 million annually in the United States [7,8]. Experts agree that enterotoxigenic *C. perfringens* infections are mainly foodborne and that meat, including poultry products, represents the most important vehicle for the transmission of the pathogen to humans [2,9,10]. Even though the first episodes of human food poisoning involving enterotoxigenic *C. perfringens* and linked to the consumption of chicken meat were reported as long as nearly 80 years ago, few research studies have looked into the presence of the pathogen in this food commodity, and even fewer have linked this food reservoir to disease outbreaks in humans [11]. Indeed, depending on the study as well as on the method used to identify and recover enterotoxigenic *C. perfringens* from retail chicken meat samples, the presence of the pathogen has been reported to vary from 0% to 24% among the chicken meat samples analyzed [12,13,14,15,16,17,18]. At the slaughter plant level, a recent study conducted by our group revealed that up to 25% of the broiler chicken carcasses sampled after chilling were positive for the presence of the *cpe* gene [19]. Although chicken meat, and more globally poultry products, have been shown to be a reservoir of enterotoxigenic *C. perfringens*, it remains unclear how and when *C. perfringens* with the potential to cause food poisoning enters the food supply chain and how it reaches the consumer’s plate. To date, many questions remain unanswered regarding the reservoirs and transmission routes of this zoonotic pathogen, thereby complicating its surveillance and control [6,20,21].

One of the reasons for the scarcity of information on the epidemiology of enterotoxigenic *C. perfringens* lies in the difficulty to isolate the pathogen. Indeed, in addition to the fact that enterotoxigenic *C. perfringens* would account for only 1% to 5% of the global *C. perfringens* population, it has been shown that the majority of these enterotoxigenic strains would also lack the *pfoA* gene encoding the theta toxin responsible for the internal zone of double hemolysis, a phenotype used to select colonies on blood agar, further impeding the recovery of this zoonotic microorganism from samples harboring a diverse and complex microbiota such as poultry meat products [22,23]. When documenting the effectiveness of different molecular approaches to detect the presence of enterotoxigenic *C. perfringens* in meat samples, Kaneko et al. highlighted the relevance of combining polymerase chain reaction (PCR) to a selective pre-enrichment step for improving the detection of the pathogen [24]. With a view to isolating enterotoxigenic *C. perfringens*, Heikinheimo et al. suggested a cultivation and detection approach specifically targeting *cpe*-carrying *C. perfringens* as well as using colony hybridization [25,26]. The aim of the current study was then to exploit the very few research results generated on the identification and isolation of enterotoxigenic *C. perfringens* in order to develop an approach that would improve the recovery of this zoonotic pathogen from broiler chicken carcass rinsates. The proposed approach includes a sequential combination of selective pre-enrichment, PCR-based detection of the *cpe* gene, classical isolation on selective growth media, and colony hybridization.

## 2. Materials and Methods

### 2.1. Sampling

Broiler chicken carcass rinsate samples used in this study were collected from two different poultry processing plants in the province of Quebec, Canada, during a previous research project conducted by our group [19]. Briefly, 16 sampling visits were conducted over a 6-month period in 2017. Five different flocks were sampled during each visit, for a total of 379 carcass rinsates recovered at five critical steps of the slaughter process [19]. After a centrifugation step (ThermoFisher Fiberlite F14-6 × 250 LE Rotor, Toronto, ON, Canada) applied on 200 mL of the carcass rinsate, the pellet was resuspended in 4 mL of buffered peptone water (Lab M Ltd., Heywood, Greater Manchester, UK) and 1 mL of the suspension was used to inoculate 9 mL of fluid thioglycolate with resazurin (FTG) (Biokar Diagnostics, Allonne, France). A PCR-based approach targeting the *cpe* gene was used on all samples after 24 h of incubation at 37 °C under anaerobic conditions (AnaeroGen Gas Generating System, Oxoid, Toronto, ON, Canada). For each PCR reaction, a reaction volume of 25 μL was used. This volume comprised 15 μL sterile water, 1× reaction buffer (10× ThermoPol reaction buffer, NEB, Canada), 0.2 μM dNTP (Bio Basic Inc., Toronto, ON, Canada), 2.5 U Taq DNA polymerase (NEB, Canada), 1 μL of each 10 μM primer, and 5 μL of template DNA as previously described by our group [19]. DNA amplification was performed using a Roche LightCycler^®^ 96 real-time PCR thermocycler (Roche Diagnostics, Laval, QC, Canada) with the following reaction conditions: a primary denaturation step at 94 °C for 2 min, followed by 35 cycles of denaturation at 94 °C for 1 min, annealing at 55 °C for 1 min, and extension at 72 °C for 1 min. Finally, a last extension step at 72 °C for 10 min was performed. Then, 10 μL of the PCR-amplified products were visualized under UV light after electrophoresis on a 1% agarose gel containing 0.01% SYBR Safe DNA gel staining (Invitrogen, Burlington, ON, Canada). The 38 samples enriched in FTG from which *cpe* was identified were centrifuged (ThermoFisher Fiberlite F14-6 × 250 LE Rotor) and the pellet was preserved in 2 mL of a powdered skim milk-based freezing medium (10% skim milk, 7.5% glucose, 10% sucrose, 1% bovine serum albumin, and distilled water) at −80 °C. It is important to mention that some samples negative for the presence of *cpe* were stored along with the *cpe*-positive samples for the purpose of the current study [19].

### 2.2. DNA Extraction

Genomic DNA extraction was carried out on the enterotoxigenic *C. perfringens* AHL 311 control strain and on the *C. perfringens* reference strain ATCC 13124 using the InstaGene matrix protocol with a 10% Chelex solution (Bio-Rad, Mississauga, ON, Canada) in water [19]. Briefly, the control and reference strains were subcultured on sheep blood agar plates (Oxoid, Toronto, ON, Canada) under anaerobic conditions at 37 °C for 24 h. Ten to 15 colonies were recovered from each agar plate, and resuspended in 1 mL of sterile distilled water. The bacterial suspension was vortexed and centrifuged at 13,000 g for 3 min. The supernatant was removed and the pellet was resuspended in 200 μL of a 10% Chelex solution. The microcentrifuge tubes were treated at 56 °C for 30 min on a hot plate, and were subsequently placed in boiling water at 100 °C for 10 min. After boiling, the bacterial suspension was vortexed and centrifuged at 13,000× *g* for 3 min. The resulting supernatant was collected and stored at −20 °C.

### 2.3. Synthesis of the Digoxigenin-Labeled DNA Probe Targeting cpe

Fragments of 333 bp from the *cpe* gene were PCR amplified as described by Heikinheimo et al. with slight modifications [25]. DNA extracted from the enterotoxigenic *C. perfringens* AHL 311 control strain was used. After amplification, the DNA was quantified by DENOVIX following the manufacturer’s instructions (DeNovix and Qubit dsDNA Assay, Biolabtech LTD, Kyiv, Ukraine) and labeled using the DIG High Prime DNA Labeling and Detection Starter kit I from Roche Diagnostics. The labeled PCR product obtained was visualized on a 1% agarose gel containing 0.01% SYBR Safe DNA gel stain as advised by the manufacturer (Invitrogen, Burlington, ON, Canada). The synthesized DNA probe was preserved at −20 °C until use to detect the *cpe* gene during the steps described below.

### 2.4. Hybridization of the Labeled Probe on Bacterial Genomic DNA and Pure Lysed Colonies of Enterotoxigenic C. perfringens AHL 311 Control Strain and C. perfringens ATCC 13124 Reference Strain

In order to validate both the specificity and sensitivity of the synthesized probe, bacterial genomic DNA and lysed colonies from *C. perfringens* AHL 311 and ATCC 13124 were used, based on the protocol described in the Roche Diagnostics Manual and according to Heikinheimo et al. [25,27]. The DNA extracted from *C. perfringens* AHL 311 and ATCC 13124 was denatured at 95 °C for 5 min, before 2 μL of the denatured DNA were placed on a nylon colony printing and plate hybridization membrane (Roche, Indianapolis, Indiana). For fixing the DNA on the nylon membranes, the latter were dried in a hybridization oven at 80 °C for 30 min.

*C. perfringens* AHL 311 and ATCC 13124 were subcultured on blood agar plates under anaerobic conditions for 24 h at 37 °C. After incubation, the grown colonies were replicated on nylon membranes by aseptically placing membranes on agar plates. Printed colonies were dried out by incubating the nylon membranes at 37 °C for 30 min. Membranes were then treated with different solutions to perform bacterial cell lysis. To carry this out, membranes were incubated in 2 mL of denaturing solution (0.5 M NaOH, 1.5 M NaCl) for 5 min, followed by 5 min of incubation in 2 mL of neutralizing solution (1.0 M Tris-HCl, 1.5 M NaCl, pH 7.4) and by a 10 min incubation step in 2 mL of 2 × saline sodium citrate (SSC) (1× SSC is 0.15 M NaCl, 0.015 M sodium citrate) (see Table 1). After bacterial cell lysis, membranes were dried at 80 °C for 30 min in a hybridization oven in order to fix the DNA.

**Figure 1:**
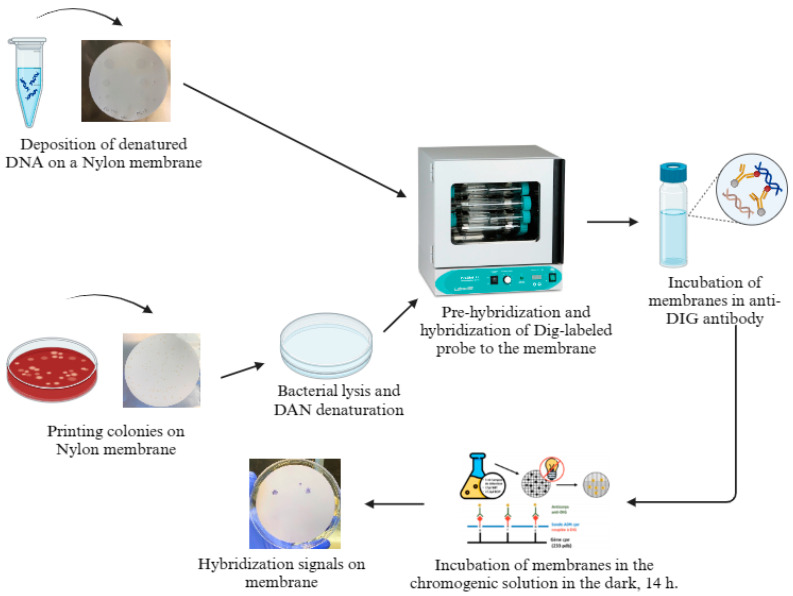
Scheme of the hybridization method. Created with BioRender.com.

Membranes were subsequently placed in a roller bottle containing the prehybridization solution (DIG Easy Hyb Granules, Roche, Indianapolis, Indiana) and then prehybridized at 42 °C for 1 h. The prehybridization solution was discarded and replaced with fresh hybridization solution containing 0.5 μL of denatured probe/mL and the incubation was continued at 42 °C for 2 h. Membranes were washed two times at room temperature, for 5 min each time, with a low stringency buffer (2 × SSC, 0.1% sodium dodecyl sulfate) added to the rolling bottles, followed by two 15 min washes at 68 °C with a high stringency buffer (0.5 × SSC, 0.1% sodium dodecyl sulphate). Membranes were then placed in a solution containing the anti-DIG antibody for 30 min, before being placed in a washing solution (kit DIG washing and blocking buffer set, Roche, Indianapolis, IN, USA) for 15 min at room temperature. This washing step was repeated two times. After washing, membranes were incubated for 5 min in a detection solution (kit DIG washing and blocking buffer set, Roche, Indianapolis, IN, USA) and then incubated for 14 h in a chromogenic solution of 5-bromo-4-chloro-3-indolylphosphate/nitro blue tetrazolium (BCIP/NBT: Sigma, St. Louis, MO, USA) in the dark at room temperature. Nylon membranes were visually analyzed to detect positive hybridization signals corresponding to the *cpe*-positive colonies after their incubation in the Tris-EDTA solution (10 mM Tris-HCl, 1 mM EDTA) as described in the DIG application manual (see Table 1 and Figure 1) [27].

### 2.5. Detection of the cpe Gene from Sterile Freezing Medium Artificially Contaminated with C. perfringens AHL 311 Control Strain Using Hydrophobic Membrane Grid Filtration and Colony Hybridization

A freezing tube containing 2.5 mL of sterile powdered skim milk-based freezing medium was artificially contaminated with 10^3^ CFU of *C. perfringens* AHL 311. Briefly, *C. perfringens* AHL 311 was grown in FTG (Biokar Diagnostics, Allonne, France) at 37 °C for 24 h in an anaerobic environment (AnaeroGen Gas Generating System, Oxoid, Toronto, ON, Canada). The bacterial culture was subjected to 10-fold serial dilutions until reaching 10^−6^ for counting. One hundred uL of the 10^−4^ dilution was used to artificially contaminate the sterile freezing medium with 10^3^ CFU of *C. perfringens* AHL 311 (see Table 1 for bacterial counts) before the freezing tube was stored at −80 °C in order to approximate the stocking conditions of the broiler carcass rinsate samples used in the present study. The artificially contaminated freezing medium was further thawed on ice before 1 mL was collected and centrifuged at (5000 rpm or 14,520× *g*) for 20 min. The pellet was then resuspended in 100 mL NaCl-Tween 80 (0.85% NaCl, Tween 80, and distilled water). After filtration through 10 hydrophobic filtration grids at a rate of 10 mL per membrane (Iso-Grid, HGMF; QA Laboratories, Toronto, ON, Canada), the grids were placed on tryptose-sulfite-cycloserine (TSC) agar plates (1% D-cycloserine and egg yolk) (Sigma-Aldrich, St. Louis, MO, USA). TSC agar plates were incubated under anaerobic conditions at 37 °C for 48 h, before plates on which a large number of isolated colonies, approximately 1250 isolated colonies, were aseptically printed on a nylon membrane, before being submitted to the hybridization steps described in Section 2.4 (see Figure 1).

### 2.6. Detection of the cpe Gene in Broiler Chicken Carcass Rinsates Artificially Contaminated with C. perfringens AHL 311 Control Strain Using Hydrophobic Membrane Grid Filtration and Colony Hybridization (HGMF-CH)

The broiler chicken carcass rinsates used were collected during a previous study conducted by our group and confirmed as *cpe* negative using a PCR-based approach as described in Gaucher et al. [19]. Tubes containing the broiler chicken carcass rinsate samples were thawed on ice before being artificially contaminated with *C. perfringens* AHL 311. For the HGMF-CH approach, three tubes were used (see Table 1). For all of these three tubes, bacterial counts of generic *C. perfringens* were established. To carry this out, 1 mL from each tube was added to 9 mL of tryptone salt (0.1% tryptone and 0.85% NaCl) and then 10-fold serially diluted to reach 10^−6^. One hundred μL of each dilution were plated on TSC agar plates (1% D-cycloserine and egg yolk) (Sigma-Aldrich, St. Louis, MO, USA) and incubated for 24 h at 37 °C under anaerobic conditions (AnaeroGen Gas Generating System, Oxoid, Toronto, ON, Canada). Subsequently, sample tubes were inoculated with 10^2^ and 10^3^ cfu of *C. perfringens* AHL 311, before being stored at −80 ℃ until analysis (see Table 1 for contamination levels). One mL from tube 3 that had been previously thawed on ice was resuspended in 200 mL 1% NaCl-Tween 80 (0.85% NaCl, Tween 80, and distilled water). After gentle agitation to uniformly resuspend the bacteria, the whole 200 mL volume was filtered at a rate of 10 mL per hydrophobic grid (ISO-GDRID; QA Laboratories, Toronto, ON, Canada) for a total of 20 grids that were subsequently placed on tryptose-sulfite-cycloserine (TSC) agar plates (LAB M, Bury, Royaume-Uni) (1% D-cycloserine and egg yolk) (Sigma-Aldrich, St. Louis, MO, USA). TSC agar plates were incubated under anaerobic conditions at 37 °C for 48 h, and the 10 agar plates on which the larger number of isolated colonies were observed were aseptically printed on a nylon membrane (Roche, Indianapolis, IN, USA) before being submitted to the hybridization steps described in Section 2.4. One mL from tubes 2 and 4 that had previously been thawed on ice was resuspended in 200 mL 1% NaCl-Tween 80. Again, after gentle agitation to uniformly resuspend the bacteria, 20 mL were filtered at a rate of 1 mL per hydrophobic grid (ISO-GDRID; QA Laboratories, Toronto, ON, Canada) for a total of 20 grids that were subsequently placed on tryptose-sulfite-cycloserine (TSC) agar plates (1% D-cycloserine and egg yolk) (Sigma-Aldrich, St. Louis, MO, USA). Bacterial growth from all 20 TSC agar plates was printed on a nylon membrane (Roche, Indianapolis, Indiana) that were subsequently submitted to the hybridization steps described in Section 2.4. For all dilution protocols, positive hybridization signals were linked to their corresponding colony on TSC agar plates and each suspect colony was subcultured on 5% sheep blood agar plates (Oxoid, Toronto, ON, Canada). The DNA from each colony was extracted using 10% Chelex (Bio-Rad, Mississauga, ON, Canada) according to the manufacturer’s instructions [24]. The *cpe* gene was PCR amplified as described by Gaucher et al. [19].

### 2.7. Detection of the cpe Gene in Broiler Chicken Carcass Rinsates Artificially Contaminated with C. perfringens AHL 311 Control Strain Using a Combination of Direct Plating and Colony Hybridization (DP-CH)

As the hydrophobic grids used for HGMF-CH approach were discontinued by the manufacturer during the development themethod by our group, an alternative protocol comprising a direct plating of the sample combined with colony hybridization (DP-CH) was also developed. Three different broiler chicken carcass rinsate samples negative for the presence of the *cpe* gene and collected during a previous study conducted by our group were identified as tubes 5, 6, and 7, and were used to validate this DP-CH approach [19]. Bacterial counts of generic *C. perfringens* were carried out as described in Section 2.6, and tubes 5 to 7 were artificially contaminated with 10^2^, 10^3^, and 10^4^ cfu of *C. perfringens* AHL 311, respectively (see Table 1). Again, different sample dilution protocols were used in order to establish the best approach according to the different levels of contamination by the enterotoxigenic *C. perfringens* selected. At the time of analysis, 1 mL from tubes 6 and 7 was recovered and diluted 1:1 in tryptone salt before being 10-fold serially diluted two times (10^−2^). A volume of one hundred μL of the 10^−2^ dilution was spread on each one of the 30 different TSC agar plates, for a total volume of 3 mL, before being incubated under anaerobic conditions for 24 h at 37 °C. Grown colonies on all 30 TSC agar plates were printed on nylon membranes that were subsequently submitted to the hybridization protocol described in Section 2.4. For analyzing tube 7 contaminated with 10^4^ cfu of *C. perfringens* AHL 311, 1 mL of the sample that had been previously thawed on ice was mixed with 9 mL of tryptone salt. The mixture was then 10-fold serially diluted until 10^−5^ and 100 uL of each dilution from 10^−2^ to 10^−5^ were individually spread on TSC agar plates that were incubated anaerobically at 37 °C for 24 h. For all sample tubes, grown bacterial colonies were printed by aseptically placing a nylon membrane directly on TSC agar plates and hybridization was performed as described in Section 2.4. After colony printing, TSC agar plates were incubated once again under anaerobic conditions for 24 h at 37 °C in order to allow further bacterial growth and facilitate recovery of *cpe*-positive colonies. Bacterial colonies linked to positive hybridization signals after application of the hybridization protocol described in Section 2.4 were retrieved from TSC agar plates, subcultured on 5% sheep blood agar plates (Oxoid, Toronto, ON, Canada) and presence of the *cpe* gene was confirmed using the PCR approach referenced above [19].

## 3. Results

### 3.1. Sampling

For the detection of the *cpe* gene from broiler chicken carcass rinsate samples artificially contaminated with *C. perfringens* AHL 311 using the HGMF-CH or the DP-CH approach, seven broiler chicken carcass rinsate samples were used, all of which tested negative for the presence of the *cpe* gene. For all of these sample tubes, counts of generic *C. perfringens* were established before the artificial contamination with *C. perfringens* AHL 311 control strain (see Table 2).

### 3.2. DNA Extraction

Three distinct DNA extractions were performed: two DNA extractions from the *cpe*-positive *C. perfringens* AHL 311 control strain that yielded 27.9 ng/µL and 33.3 ng/µL of DNA and one DNA extraction from the *cpe*-negative ATCC 13124 *C. perfringens* reference strain yielding 28.0 ng/µL of DNA. Ten ng of genomic DNA from AHL 311 were added to 50 μL of DIG kit reagents as recommended by the manufacturer, and 2 μL of this 50 μL suspension were used for DNA–DNA probe hybridization according to the Roche Manual [27].

### 3.3. Synthesis of the Digoxigenin-Labeled DNA Probe Targeting cpe

**Figure 2:**
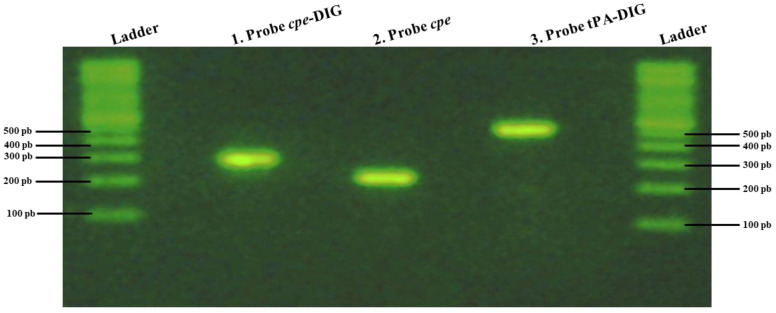
PCR typical results obtained on 1% agarose gels after electrophoresis following the synthesis of a digoxigenin (DIG)-labeled DNA probe (probe 2 in this case) used in this study. 1. DIG-labeled probe. 2. Unlabeled positive control, which corresponds to the amplification of the *cpe* gene without DIG. 3. Labeled positive control which corresponds to the labeled probe that recognizes tissue plasminogen activator (tPA) sequences used as a positive control (according to the Roche Diagnostics protocol).

DNA probes were synthesized by PCR, incorporating the digoxigenin-substituted ribonucleotide dig-UTP (see Figure 2). For the labeled-DNA probe synthesis, seven distinct assays giving the same probe with different concentrations varying from 4.57 ng/µL to 54.8 ng/µL were performed (see Table 3). For each hybridization assay, 25 ng of DIG-labeled DNA probe/mL of hybridization buffer was used according to the Roche guidelines [27].

### 3.4. Hybridization of the Labeled Probe and the cpe Gene from Bacterial Genomic DNA or Pure Lysed Bacterial Cells from C. perfringens AHL 311 Control Strain and C. perfringens Reference Strain ATCC 13124

**Figure 3:**
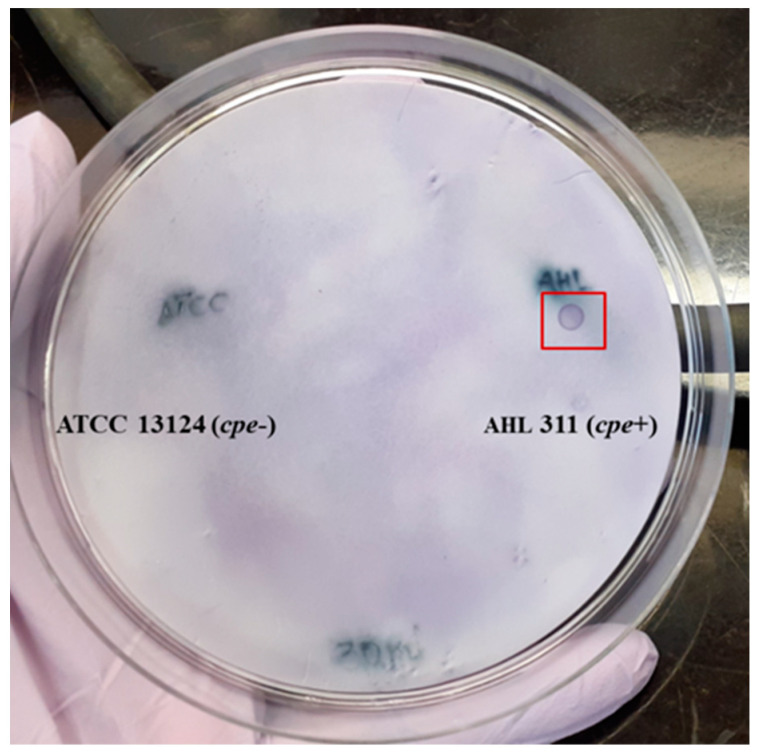
Nylon membrane after hybridization between the labeled probe and the DNA from *C. perfringens* control strains used in this study (AHL 311 and ATCC 13124); positive hybridization signals were observed for AHL 311 only. Red square is showing the DNA demonstrating a positive hybridization signal.

**Figure 4:**
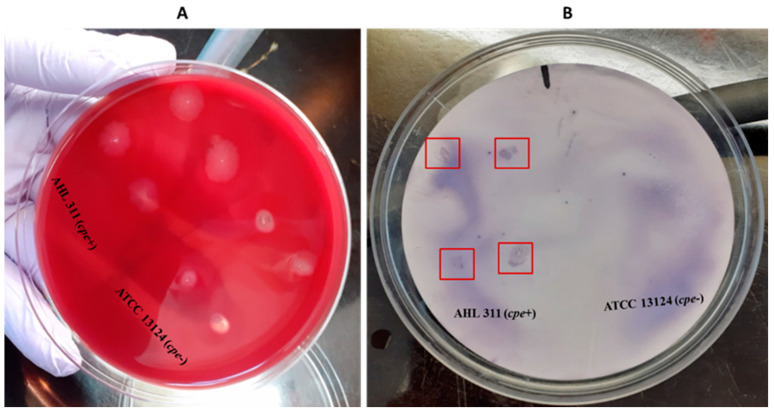
Hybridization from pure lysed colonies of *C. perfringens* reference strains positive and negative for the *cpe* gene (AHL 311 and ATCC 13124). (**A**) Bacterial growth on a blood agar. (**B**) Nylon membrane after hybridization between the labeled probe and lysed colonies of *C. perfringens* reference strains used in this study; positive hybridization signals were observed from AHL 311 lysed colonies, whereas no hybridization signal was generated for ATCC 13124. Red squares are showing colonies demonstrating a positive hybridization signal.

Hybridization of the labeled-DNA probe was performed to confirm the specificity of the probe. Four separate hybridization tests were performed: Two with the *C. perfringens* AHL 311 genomic DNA and two with pure lysed bacterial colonies from this same control strain. Positive hybridization signals visually appeared as small purple dots on nylon membranes. These signals were detected for all membranes on which DNA samples and lysed colonies of *C. perfringens* AHL 311 were dropped or printed, respectively. Conversely, no positive hybridization signal was observed for membranes on which DNA or lysed colonies of the reference strain *C. perfringens* ATCC 13124 were present (see Figure 3 and Figure 4).

### 3.5. Detection of the cpe Gene from Sterile Freezing Medium Artificially Contaminated with C. perfringens AHL 311 Using a Hydrophobic Grid Membrane Filtration and Colony Hybridization (HGMF-CH) Approach

**Figure 5:**
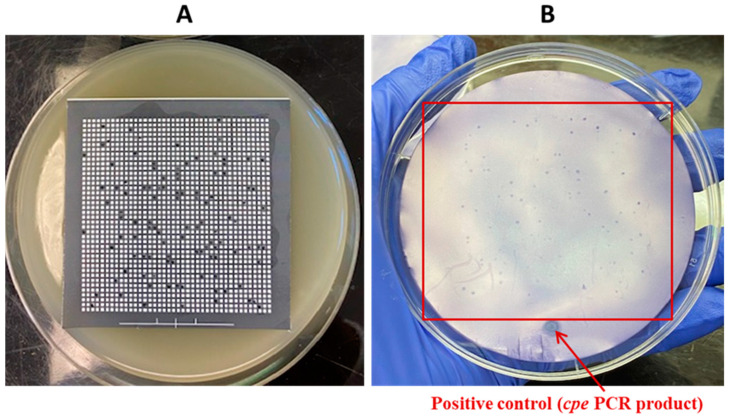
Hybridization from lysed colonies grown from a sterile freezing medium artificially contaminated with *C. perfringens* AHL 311. (**A**) Bacterial growth of *C. perfringens* AHL 311 from sample tube 4 on one of the 10 hydrophobic grids laid on TSC agar plates. (**B**) Positive hybridization signals from *cpe*-positive colonies of *C. perfringens* AHL 311 added to tube 4 containing a sterile freezing medium filtered on hydrophobic grids incubated on TSC agar plates and printed on nylon membranes. The positive control corresponds to the *cpe* PCR product. Red square is showing colonies demonstrating a positive hybridization signal.

Assays using the sterile freezing medium (tube 4, see Table 2) artificially contaminated with 2.5 × 10^3^ cfu of *C. perfringens* AHL 311 control strain were performed. After filtration and incubation of the hydrophobic grids, a total of 980 colonies were counted from the 10 TSC agar plates used and hybridization signals were observed from these colonies on all membranes. Positive hybridization signals were not counted at this step even though the estimation of positive hybridization signals averaged the total number of colonies. Figure 5 shows one of the 10 TSC agar plates and its corresponding nylon membrane revealing positive hybridization signals.

### 3.6. Detection of the cpe Gene from Broiler Chicken Carcass Rinsates Artificially Contaminated with C. perfringens AHL 311 Control Strain Using Hydrophobic Grid Membrane Filtration and Colony Hybridization

**Figure 6:**
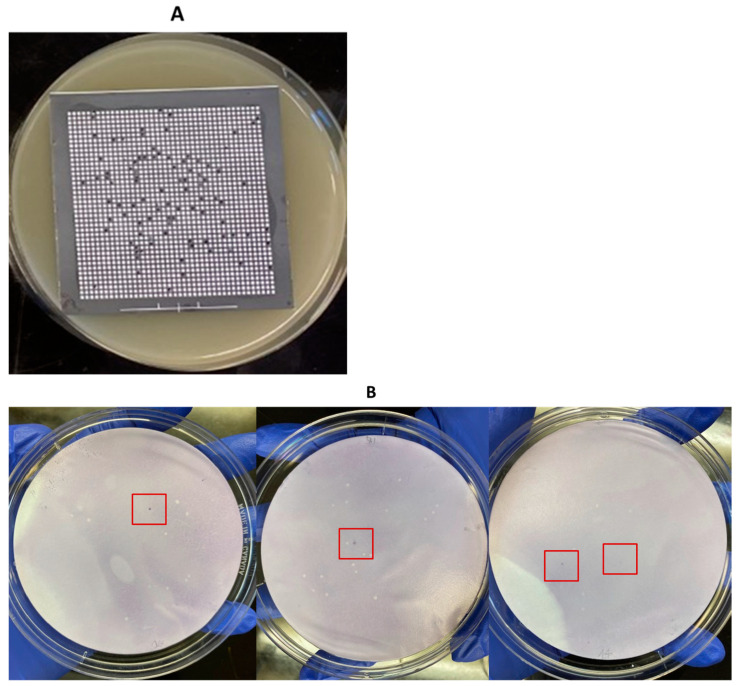
Application of the HGMF-CH method using samples of broiler chicken carcass rinsates artificially contaminated with *C. perfringens* AHL 311 (tube 1, Table 2). (**A**) *C. perfringens* growth on a hydrophobic grid incubated on TSC agar plates after the filtration of a broiler chicken carcass rinsate sample artificially contaminated with *C. perfringens* AHL 311 (corresponding to sample tube 1). (**B**) Colonies showing positive hybridization signals on a nylon membrane printed from TSC agar plates with *C. perfringens* growth (**A**). Red squares are showing colonies demonstrating a positive hybridization signal.

**Figure 7:**
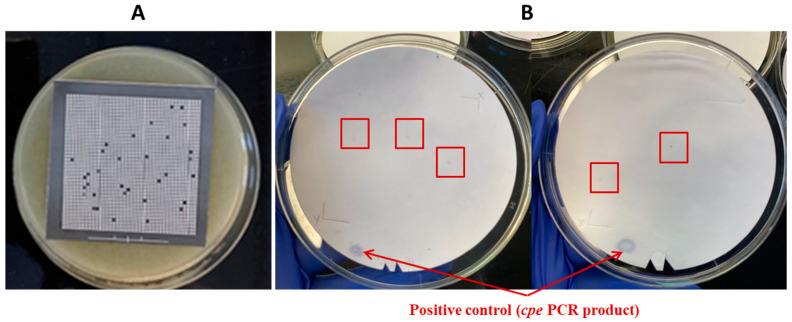
Application of the HGMF-CH method using samples of broiler chicken carcass rinsates artificially contaminated with *C. perfringens* AHL 311 (tube 3, Table 2). (**A**) *C. perfringens* growth on a hydrophobic grid incubated on TSC agar plates after the filtration of a broiler chicken carcass rinsate artificially contaminated with *C. perfringens* AHL 311. (**B**) Positive hybridization signals on a nylon membrane printed from TSC agar plates with *C. perfringens* growth (**A**) were filtered. The positive control (*cpe* PCR product) appears on the nylon membrane. Red squares are showing colonies demonstrating a positive hybridization signal.

**Figure 8:**
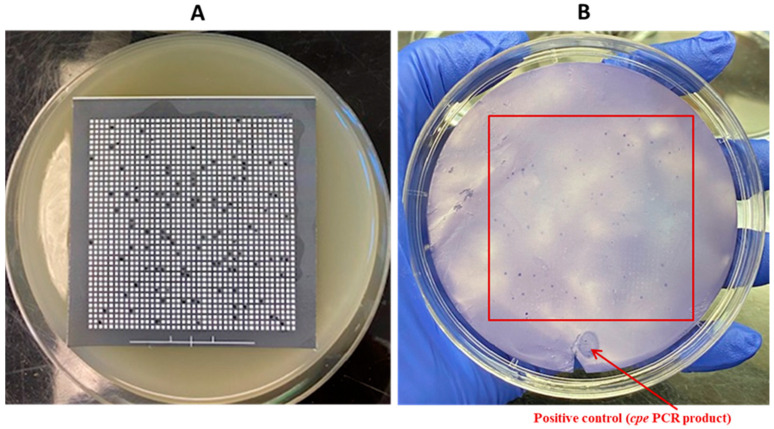
Application of the HGMF-CH method using samples of broiler chicken carcass rinsates artificially contaminated with *C. perfringens* AHL 311 (tube 2, Table 2). (**A**) *C. perfringens* growth on a hydrophobic grid incubated on TSC agar plates after the filtration of a broiler chicken carcass rinsate sample artificially contaminated with *C. perfringens* AHL 311. (**B**) Colonies showing positive hybridization signals on a nylon membrane printed from TSC agar plates with *C. perfringens* growth (**A**) were filtered. The positive control (*cpe* PCR product) appears on the nylon membrane. Red square is showing colonies demonstrating a positive hybridization signal.

**Figure 9:**
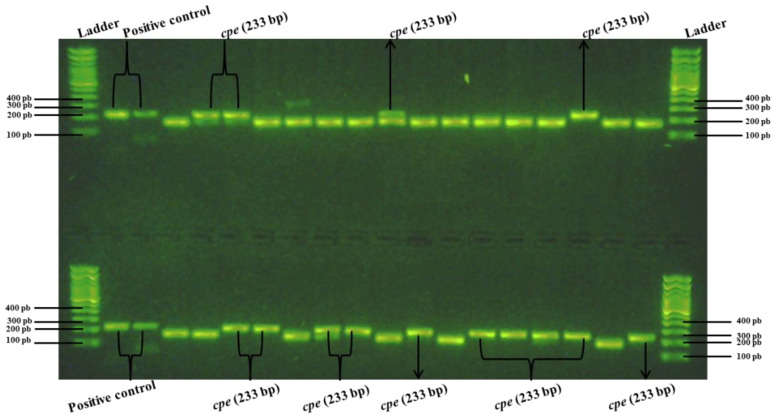
PCR analysis of the suspect *C. perfringens* colonies subcultured on sheep blood agar plates after recovery from TSC agar plates following the detection of corresponding positive hybridization signals on nylon membranes. The 233 bp fragments correspond to *C. perfringens* enterotoxin-encoding gene, *cpe* (tube 2, see Table 2).

The HGMF-CH approach was applied to three tubes (tubes 1 to 3) containing broiler chicken carcass rinsates negative for the presence of the *cpe* gene and artificially contaminated with the *C. perfringens* control strain AHL 311 (see Table 2). For sample tubes 1 and 3 that were submitted to the same dilution and filtration protocol, 20 nylon membranes were printed from hydrophobic grids. A total of 275 bacterial colonies were observed, among which four positive hybridization signals were identified for sample tube 1 (Figure 6). The printing of 20 TSC agar plates from the filtration of sample tube 3 for which the concentration of generic *C. perfringens* was similar to the concentration of this bacterial population found in sample tube 2 yielded 998 colonies from which five hybridization signals could be observed (Figure 7). For sample tube 2, for which 10 mL on 20 nylon membranes and of which only 10 membranes were printed from hydrophobic grids, the hybridization signals were detected for this tube. However, neither colony counts nor hybridization signals were performed for this tube (Figure 8). The PCR result from tube 2 revealed that 14 of 32 randomly selected *C. perfringens* isolates tested were found to carry the *cpe* gene (Figure 9). The PCR results corresponding to 200 bp bands were submitted to sequencing and were confirmed to be sequences corresponding to enterococci (Figure 9).

### 3.7. Detection of the cpe Gene from Broiler Chicken Carcass Rinsates Artificially Contaminated with C. perfringens AHL 311 Using a Combination of Direct Plating and Colony Hybridization (DP-CH)

**Figure 10:**
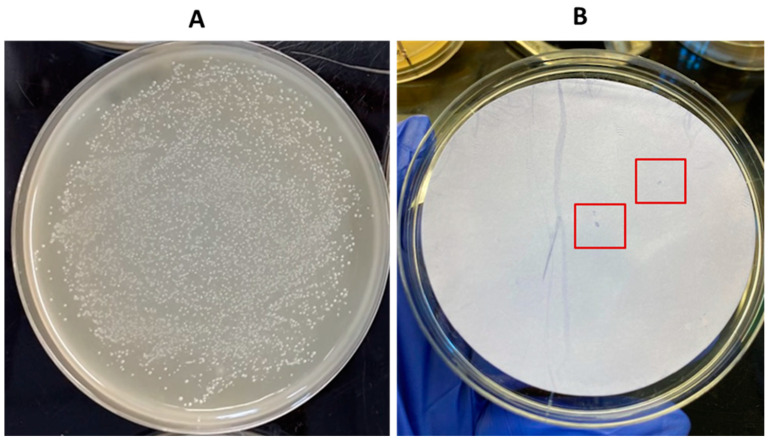
Application of the DP-CH method using samples of broiler chicken carcass rinsates artificially contaminated with *C. perfringens* AHL 311 (tube 5, Table 2). (**A**) Bacterial growth on a TSC agar plate obtained from the analysis of tube 5. (**B**) Positive hybridization signals from colonies printed from the corresponding TSC agar plate (see in (**A**)) (tube 5, see Table 2). Red squares are showing colonies demonstrating a positive hybridization signal.

**Figure 11:**
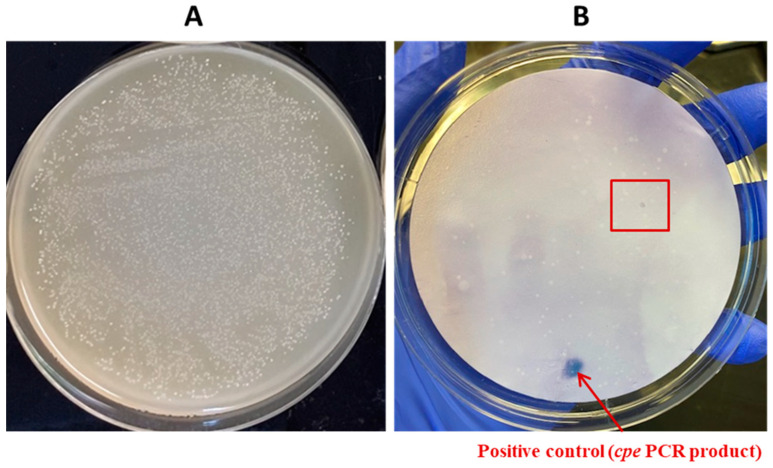
Application of the DP-CH method using samples of broiler chicken carcass rinsates artificially contaminated with *C. perfringens* AHL 311 (tube 6, Table 2). (**A**) Bacterial growth on a TSC agar plate obtained from the analysis of tube 6. (**B**) Positive hybridization signals from colonies printed from the corresponding TSC agar plate (see in (**A**)) and from the positive control (*cpe* PCR product) (tube 6, Table 2). Red square is showing colonies demonstrating a positive hybridization signal.

**Figure 12:**
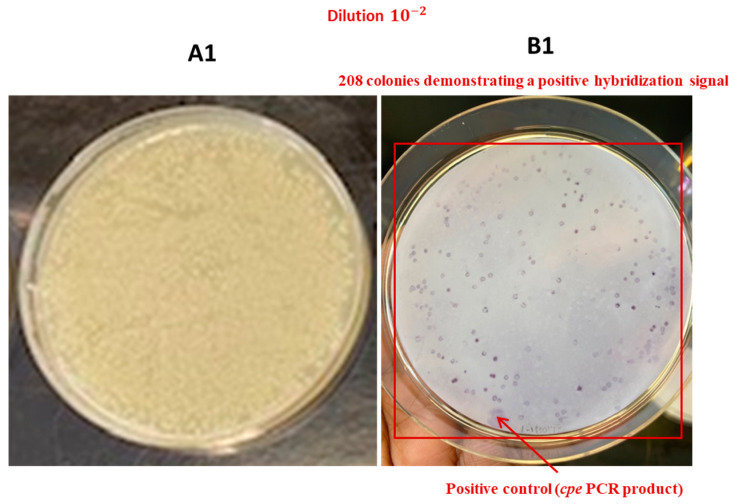

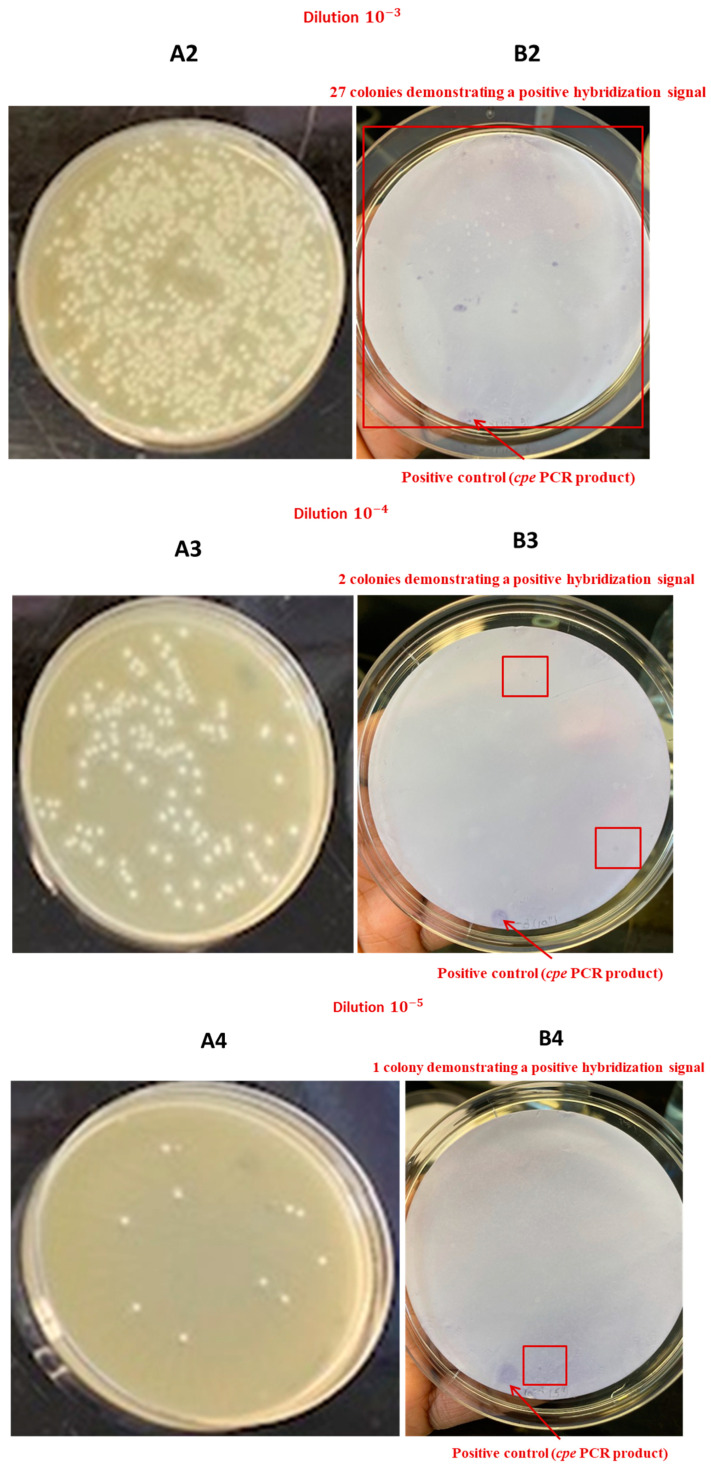
Application of the DP-CH method using samples of broiler chicken carcass rinsates artificially contaminated with *C. perfringens* AHL 311 (tube 7, Table 2). (**A1**–**A4**) Bacterial growth on a TSC agar plate obtained from the analysis of tube 7. (**B1**–**B4**) Positive hybridization signals from colonies printed from the corresponding TSC agar plate (see in (**A1**–**A4**)) and from the positive control (*cpe* PCR product). Red squares are showing colonies demonstrating a positive hybridization signal.

**Figure 13:**
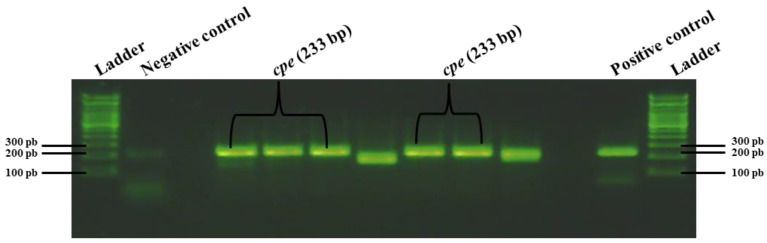
PCR analysis of the suspect *C. perfringens* colonies subcultured on sheep blood agar plates after recovery from TSC agar plates following the detection of corresponding positive hybridization signals on nylon membranes. Fragments of 233 bp correspond to *C. perfringens* enterotoxin-encoding gene, *cpe* (tube 7, see Table 2).

In order to validate the usefulness of the hybridization approach for the recovery of enterotoxigenic *C. perfringens* from broiler chicken carcass rinsate samples without resorting to the hydrophobic filtration grids, three sample tubes, tubes 5 to 7, containing broiler chicken carcass rinsates negative for the presence of the *cpe* gene and artificially contaminated with the *C. perfringens* control strain AHL 311 were used, as described in Section 2.7 and Section 3.1. Similar to the HGMF-CH approach, the DP-CH method was associated with positive hybridization signals for all sample tubes. Membranes printed from the 30 TSC agars plated from tube 5 artificially contaminated with 8.5 × 10^2^ cfu of *C. perfringens* AHL 311 revealed positive hybridization signals for two of a total of colonies that were quite numerous to be counted (see Figure 10). For tube 6 that was inoculated with 1.43 × 10^3^ cfu of AHL 311, a positive hybridization signal was observed for one of a total of colonies that were again quite numerous to be counted (see Figure 11), and finally, the analysis of tube 7 that was artificially contaminated with 8.5 × 10^4^ cfu of *C. perfringens* AHL 311 revealed positive hybridization signals for 239 of 777 grown colonies (see Figure 12). For this latter sample tube, a PCR approach confirmed the presence of the *cpe* gene in five of seven presumptive *cpe*-positive isolates analyzed from TSC agar plates. The two bands corresponding to 200 bp were submitted to sequencing and were confirmed to be sequences corresponding to enterococci (see Figure 13).

## 4. Discussion

Enterotoxigenic *C. perfringens* remains one of the three zoonotic bacterial pathogens most frequently incriminated in foodborne disease outbreaks in many industrialized countries, along with *Salmonella* and *Campylobacter* [28]. Many elements support the fact that enterotoxigenic *C. perfringens* could have a greater contribution to the burden of foodborne illnesses worldwide. This pathogen is widely distributed in a diversity of environments including soils and the digestive tract of all warm-blooded animals and humans. The major challenges associated with the isolation of the enterotoxigenic subpopulation of this bacterial species are mainly due to the absence of specific phenotypic attributes enabling its rapid recovery and identification, and to the relatively short duration of the gastrointestinal disease it causes [6,7]. Foodstuffs of animal origin have long been reported as the main reservoir contributing to human exposure to foodborne *C. perfringens*. However, the epidemiology of this pathogen still remains poorly described due to the difficulty of recovering enterotoxigenic strains [6,9,29]. A previous study conducted by our group revealed that broiler chicken meat could represent an important source of enterotoxigenic *C. perfringens* based on the prevalence of *cpe*-positive broiler chicken carcasses identified at the end of the slaughter process. As the isolation and molecular characterization of the *C. perfringens* strains harboring this *cpe* gene detected on these broiler chicken carcasses would help better define poultry as a reservoir of this foodborne microorganism, the aim of the current work was to develop an isolation protocol allowing for the specific recovery of enterotoxigenic *C. perfringens* strains from carcass rinsates which were found to be *cpe* positive.

Some authors aiming to describe the role of poultry meat as a reservoir of this zoonotic pathogen may have underestimated its prevalence when not using a PCR-based screening step on pre-enriched meat samples and by investigating the presence of *cpe* in a limited number of selected colonies grown on agar plates [12,13,15,17]. The HGMF-CH approach comprises filtering samples before printing the grown colonies on a nylon membrane that will be submitted to hybridization of the bacterial denaturated DNA with a probe specifically designed as a complementary DNA sequence. Colony hybridization was first described nearly 50 years ago and was shown at that time to facilitate the recovery of microorganisms bearing specific DNA or gene fragments [30,31]. Some researchers used this approach for the detection of *Listeria monocytogenes, Escherichia coli (VTEC),* and *Campylobacter jejuni* [32,33,34]. Heikinheimo et al. were the first to apply colony hybridization to a hydrophobic grid membrane filtration approach to isolate enterotoxigenic *C. perfringens*, and thus to better document the role of humans in the carriage and transmission of this zoonotic pathogen [25,26]. As reported by this research group, the 333 bp DNA probe synthesized to detect a fragment of the highly conserved *C. perfringens cpe* gene in the current study was revealed to be 100% sensitive when tested on both pure bacterial DNA and cultures of the *C. perfringens* control strains used [25]. The sensitivity of the labeled probe was further confirmed with the assay performed on sample tube 4 from which every single colony grown on the hydrophobic grid laid on TSC agar plates was attributed to a positive hybridization signal, thereby also confirming the sterility of the medium used. This test involving sample tube 4 resulted in the growth of a slightly lower number of 980 presumptive *C. perfringens* colonies compared to the expected 1250 CFU according to the contamination level used. This discrepancy highlights a loss that could be attributed to various factors related to the methodology such as a certain permeability of the hydrophobic grids which was subsequently linked to the withdrawal of the product from the market by the manufacturer.

In the present study, sample tubes 1 to 3 were used to validate both the dilution filtration protocol of the HGMF-CH approach when applied to broiler chicken carcass rinsate samples. For both sample tubes 1 and 3, the bacterial suspension was filtered through 20 hydrophobic grids; therefore, establishing the detection limit at 3.2 × 10^−4^ of *cpe*-positive *C. perfringens*, which nearly corresponded to the ratio between both generic and enterotoxigenic *C. perfringens* populations used for this tube (1/38,000). As five positive hybridization signals could be observed on the 20 membranes printed from the analysis of this sample tube, we could then estimate the limit to detect one *cpe*-positive colony to be as low as 1.9 × 10^−5^, approximating the value reported by Heikinheimo et al. [25]. However, the low number of 998 grown colonies observed on the TSC agars plated from this sample tube again probably reflected a suboptimal impermeability of the filtration grids, potentially jeopardizing the recovery of the enterotoxigenic *C. perfringens* colonies if this ratio would have been maintained during filtration. When considering the contamination levels used for *C. perfringens* AHL 311, the results obtained in the current study highlight the suitability and effectiveness of the dilution filtration protocol to be applied to field samples corresponding to those used for the isolation of *cpe*-positive *C. perfringens*. Indeed, the detection limit of a molecular-based approach such as conventional PCR is recognized to vary between 10^2^ and 10^4^ copies of genes, corresponding to the contamination levels used for sample tube 4 [35]. When investigating the usefulness of the same dilution filtration protocol on field samples contaminated with lower levels of enterotoxigenic *C. perfringens* and that would correspond to the lower detection range for a conventional PCR detection approach, the assay performed with sample tube 2 was associated with four positive hybridization signals, again establishing the actual detection limit around 2 × 10^−5^, similar to the limit identified with sample tube 4, and confirming the potential of the approach proposed for the recovery of *cpe*-positive *C. perfringens* from less contaminated broiler chicken carcass samples that would be submitted to a pre-enrichment step beforehand. These observations show that this approach is effective in detecting enterotoxigenic *C. perfringens* for which the ratio with generic strains of this same bacterial species is between 1.9 and 2 × 10^−5^, corresponding to 1 CFU/mL after enrichment of the initial sample.

Similar concentrations of the control strain *C. perfringens* AHL 311 were used for the contamination of tubes 5 and 6 in order to establish the applicability of a hybridization approach to a direct plating of broiler chicken carcass rinsate samples following the discontinuation of the hydrophobic filtration grids. The dilution plating protocol tested established the theoretical detection limit at 1 and 3.5 *cpe*-positive colonies for sample tubes 5 and 6, respectively, according to the contamination levels used. The results obtained from the direct plating of a total of 30 TSC agar plates stressed the importance of considering the screening of a considerable number of nylon membranes printed out from these agar plates as only a single positive hybridization signal could be generated from the analysis of sample tube 6. On the other hand, the approach considering dilution steps and the plating of a restricted volume of a specific bacterial suspension brings into play a random aspect of the procedure that could be associated with increases in the sensitivity of the approach, which was two-fold for the analysis of sample tube 6 in the current study. Results from a previous study conducted by our group showed the importance of using a primary discriminatory PCR step to confirm the *cpe* status of a sample as the presence of enterotoxigenic *C. perfringens* on broiler chicken carcasses can be highly variable along the slaughter line [19]. However, when positivity rates reach more than 25% as it was also documented during this previous study, the large number of nylon membranes and hybridization assays can be a limiting factor when considering the financial and time aspects of this isolation procedure. Sample tube 7 was used to validate if a direct plating approach limited the number of agar plates to be spread, and therefore, the number of nylon membranes to be submitted to hybridization could be used for the recovery of *cpe*-positive *C. perfringens* from broiler chicken carcass rinsates. The dilution plating protocol was designed according to a contamination level by *C. perfringens* AHL 311 established at 10^5^, with a detection limit approaching 1 *cpe*-positive *C. perfringens* for the 10^−4^ dilution, and being established at 8, 85, and 850 *cpe*-positive colonies for the 10^−3^, 10^−2^, and 10^−1^ dilutions, respectively. On any of the nylon membranes printed out from the TSC agar plates, a higher number of positive hybridization signals were identified, this number matching to 2, 27, and 208 *cpe*-positive colonies on agar plates that corresponded to the 10^−3^, 10^−2^, and 10^−1^ dilutions, respectively. The results show that the direct plating approach combined with colony hybridization proved to be sensitive for the detection of enterotoxigenic strains of *C. perfringens*, with a theoretical detection limit varying between 1 and 3.5 *cpe*-positive *C. perfringens* colonies/mL. However, this protocol has one major drawback. As it can represent a clear benefit when the ratio of enterotoxigenic *C. perfringens* to generic *C. perfringens* is approximately 1, leaving an almost equal space for the growth of both subpopulations, it can be more challenging when *cpe*-positive strains are less abundant and must be recovered from a bacterial mat covering the agar plate, justifying additional dilutions and further reducing this ratio. Based on the results obtained, we can hypothesize that a ratio of enterotoxigenic *C. perfringens* to generic *C. perfringens* of 1:10 or even 1:100 would significantly decrease the chances of recovering *cpe*-positive strains from a complex sample such as broiler chicken carcass rinsates. In these circumstances, the plating of additional agar plates and a subsequent hybridization performed on more nylon membranes could assist. In the current study, different protocols were validated, including various dilution, filtration, and plating approaches. Results confirm that colony hybridization allows for the rapid identification of *cpe*-positive *C. perfringens* in a complex sample, as previously reported [25]. Different ratios of enterotoxigenic *C. perfringens* to generic *C. perfringens* were tested and suggest that the application of a pre-enrichment step, allowing the *cpe*-positive *C. perfringens* population to reach the concentrations corresponding to the lower reference of the range of values established for the detection limit of a conventional PCR approach, should be part of a more global approach for the recovery of enterotoxigenic *C. perfringens* from broiler chicken carcasses.

## 5. Conclusions

This study describes the use of a hydrophobic grid filtration or a direct plating approach combined with colony hybridization for the detection of enterotoxigenic *C. perfringens* in broiler chicken carcass rinsate samples. The results obtained confirm that this approach allows for the recovery of *cpe*-positive isolates from these samples, with a detection limit aligning to probable contamination ratios observed on broiler chicken carcasses at the slaughter plant level. We believe this work will help improve the study of the epidemiology of this zoonotic pathogen. The approach developed and described is however not currently a routinely applicable method due to the efforts required. The evaluation of a shortened protocol limiting the number of agar plates and hybridization assays would be worth considering once future studies will have documented the actual levels of contamination of broiler chicken carcasses by both *cpe*-positive and generic *C. perfringens*.

## Figures and Tables

**Table 1:** Hybridization steps, buffers, and incubation times.

Hybridization Step	Buffers	Time/Temperature
Bacterial lysis and DNA denaturation	Denaturing solution: 0.5 M NaOH, 1.5 M NaCl	5 min/Room T.
	Neutralizing solution: 1.0 M Tris-HCl, 1.5 M NaCl, pH 7.4	5 min/Room T.
	2 × saline sodium citrate (SSC): 0.3 M NaCl, 30 mM sodium citrate	10 min/Room T.
Pre-hybridization	Easy Buffer hyb	1 h/42 °C
Hybridization of the probe	Easy Buffer hyb and probe	2 h/42 °C
Membrane washing	Stringency buffer: 2 × SSC, 0.1% SDS	2 × 5 min/Room T.
	Stringency buffer: 0.5 × SSC, 0.1% SDS	2 × 15 min (68 °C)
	Washing buffer B1 (kit DIG washing and blocking buffer set)	5 min/Room T.
Membrane blockage	Blocking buffer: B2 and B3 (kit DIG washing and blocking buffer set)	30 min/Room T.
Antibody binding	Anti-DIG antibody and blocking buffer B2 and B3 (kit DIG washing and blocking buffer set)	30 min/Room T.
Membrane washing	Washing buffer B1 (kit DIG washing and blocking buffer set)	15 min/Room T.
Signal detection	Detection buffer B4: kit DIG washing and blocking buffer set	5 min/Room T.
	Detection buffer B4 + chromogenic solution (NBT/BCIP)	14 h/Room T. in the dark
Interruption of the chromogenic reaction	TE buffer (Tris-EDTA)	5 min/Room T.

DNA: deoxyribonucleic acid; NaOH: sodium hydroxide; NaCl: sodium chloride; SSC: saline sodium citrate; SDS: sodium dodecyl sulfate; Tris-HCl: tris-hydrochloride; hyb: hybridization; DIG: digoxigenin; NBT: nitro blue tetrazolium; BCIP: 5-bromo-4-chloro-3-indolyl-phosphate; Tris-EDTA: tris-ethylenediaminetetraacetic; T: temperature.

**Table 2:** Sample tubes used and their respective bacterial concentrations for generic *C. perfringens* and enterotoxigenic *C. perfringens* AHL 311 control strain used for the development of the HGMF-CH and DP-CH approaches in the current study.

	Sample Tube Identification	Bacterial Counts (cfu/mL)	
		Generic *C. perfringens*	Enterotoxigenic *C. perfringens* AHL 311 Control Strain
HGMF-CH approach	Tube 1	4.3 × 10^7^	8.5 × 10^2^
	Tube 2	1.3 × 10^6^	5.8 × 10^3^
	Tube 3	6.5 × 10^7^	1.71 × 10^3^
	Tube 4	Sterile freezing medium	2.5 × 10^3^
DP-CH approach	Tube 5	4.3 × 10^7^	8.5 × 10^2^
	Tube 6	1.76 × 10^5^	1.43 × 10^3^
	Tube 7	5.6 × 10^7^	8.5 × 10^5^

HGMF-CH: hydrophobic grid membrane filtration-colony hybridization; DP-CH: direct plating and colony hybridization; cfu: colony forming unit; mL: milliliter.

**Table 3:** Synthesized probes and their respective concentration.

Probes Synthesized in the Current Study	Probe Concentration (ng/µL)
Assay 1	4.57
Assay 2	25.8
Assay 3	35.2
Assay 4	46.2
Assay 5	37.6
Assay 6	51.9
Assay 7	54.8

ng: nanogram; µL: microliter.

## Data Availability

The data presented in this study are all available in the current manuscript.
